# Communities' awareness of afforestation and its contribution to the conservation of lizards in Dodoma, Tanzania

**DOI:** 10.1038/s41598-022-27268-7

**Published:** 2022-12-30

**Authors:** Kelvin Ngongolo, Mhuji Kilonzo

**Affiliations:** Department of Biology, College of Natural and Mathematical Sciences, University of Dodoma, Dodoma, Tanzania

**Keywords:** Ecology, Zoology, Ecology, Environmental sciences, Environmental social sciences

## Abstract

Afforestation is providing the remedy for deforestation, which is among the greatest challenges of biodiversity conservation in Tanzania. Efforts for afforestation are taking place in Dodoma, which are anticipated to have social and ecological positive effects. This study provides information on the perception of local communities towards afforestation and how afforestation can harbor other wildlife species like lizards. A semi-structured questionnaire was used to collect information from respondents who were chosen at random in the afforestation area. Pitfalls and direct observation under constrained time intervals were used to sample lizards in two categories of vegetation (afforested and non-afforested areas; n = 1040 samples). Awareness of afforestation was significantly affected by age group (*P* < 0.005) and nature of course taking. In this case, those who studied natural courses like biology, forest, and aquatic science were more aware of afforestation and they had a likelihood of being involved in afforestation programs (*P* < 0.05). Eight species of lizards were identified in the study area where seven species were found in afforested areas while three were found in non-afforested areas, where *Agama lionotus* was the dominant species*.* The diversity of lizards was higher in afforested areas (Shannon Weiner index H = 1.37) than in non-afforested areas (Shannon Weiner index H = 0.99). More afforestation program awareness and conservation education are required to ensure the sustainability of afforestation efforts in Dodoma. Afforestation showed a significant contribution to the conservation of lizards. Lizards can be used as good indicator species to understand and monitor the success of afforestation.

## Introduction

Afforestation is the establishment of a forest or stand of trees in an area where there was no previous tree cover for the purpose of improving and modifying the ecosystem in a particular area for specific requirements. The requirements for afforestation could be socio-economic need, ecological functioning, or conservation purposes. Other studies have shown that afforestation plays a role in the regulation of temperature^1–3^. For instance, a study by^1^ showed that, afforestation reduced temperatures by 0.45–0.25 °C over a period of 20 years. Also, findings from the Amazon Forest revealed that, forest occurrences are triggered by the water vapour generated from the forest^4^. Furthermore, afforestation has been reported to have socio-economic and ecological benefits such as the introduction of new species, food, firewood, building poles, thatching graces, carbon-sequestration, beekeeping, and provision of grazing areas^5^.

Dodoma City has started a regreening program which intends to improve the semi-arid areas by planting trees which can adapt well in these areas. It was launched in 2017 by government^6^. The ideas have been absorbed by different institutions such as the World-wide Fund for Nature (WWF), Tanzania Forest Services (TFS), and the University of Dodoma (UDOM)^6^. The University of Dodoma, being a key stakeholder in afforestation, has dedicated its resources in terms of human and material resources to the afforestation program within UDOM campuses. It has been reported that more than 1000 cashew nut trees are planted in this area^7^. Regardless of the effort taking place in afforestation in UDOM, less is known about how these afforestation projects contribute to the conservation of wildlife species such as lizards.

Forests provide socio-economic benefits to local communities adjacent to them^7,8^. Likewise, the afforested areas are anticipated to provide benefits to local communities who are surrounding the area where afforestation is taking place^9–12^. The benefits from the forest can be provided by wildland fruits, firewood, medicinal herbs, habitat for endangered species, income generation and employment^9,13^. Also, trees from forests help reduce air pollution, fight the atmospheric greenhouse effect, conserve water and reduce soil erosion, save energy, modify the local climate, increase economic stability, reduce noise pollution, create wildlife and plant diversity, and increase property values^14^. Awareness of the benefits accrued from afforestation by the local communities is important. This provides an opportunity and motivates the local communities to get involved in conservation of the restored areas. The impact of the afforestation program on the local communities adjacent to the afforested areas has not been documented.

Afforestation activities are supposed to be monitored for their success or failure. Lizards have been shown to be sensitive to habitat changes^15^. Dodoma, being a semi-arid area, requires species which are adaptive and can be used as indicator species such as reptiles and insects^16^. Although afforested areas will act as habitats for habitats, the lizards can act as indicators for monitoring the success of the afforested areas. A study in the Sab-Sahara areas revealed that lizards are good bioindicators for monitoring biodiversity health, particularly in semi-arid areas like Dodoma^17^. Their abundance and diversity are anticipated to show the ecosystem health of a particular area. For instance, a study in Andean Ecuador showed that human-induced habitat changes and elevation had significant effects on the abundance and diversity of lizards^18^. Despite the sensitivity and importance of lizards in monitoring biodiversity health, little is known about how the afforestation program affects the diversity and abundance of lizards in Dodoma, specifically at the University of Dodoma. Furthermore, the community's awareness of the ongoing deforestation at the University of Dodoma has not been thoroughly investigated. Understanding the effects of afforestation on lizard abundance and diversity in Dodoma will help with biodiversity conservation strategies. This will also aid in the use of lizards as good bioindicators in monitoring the success of the afforested area. The increased awareness of communities in the area will allow us to plan the development of conservation education for the long-term conservation of the afforested areas.

The purpose of this study was to better understand community awareness and association with the Dodoma afforestation effort. Study stressed the need to assess the contribution of the afforestation program to the conservation of wildlife species, specifically lizard diversity. Furthermore, we summarized different species of plants which are involved in restoration and assessed the awareness of communities about the program.

## Materials and methods

### Study area

**Figure 1:**
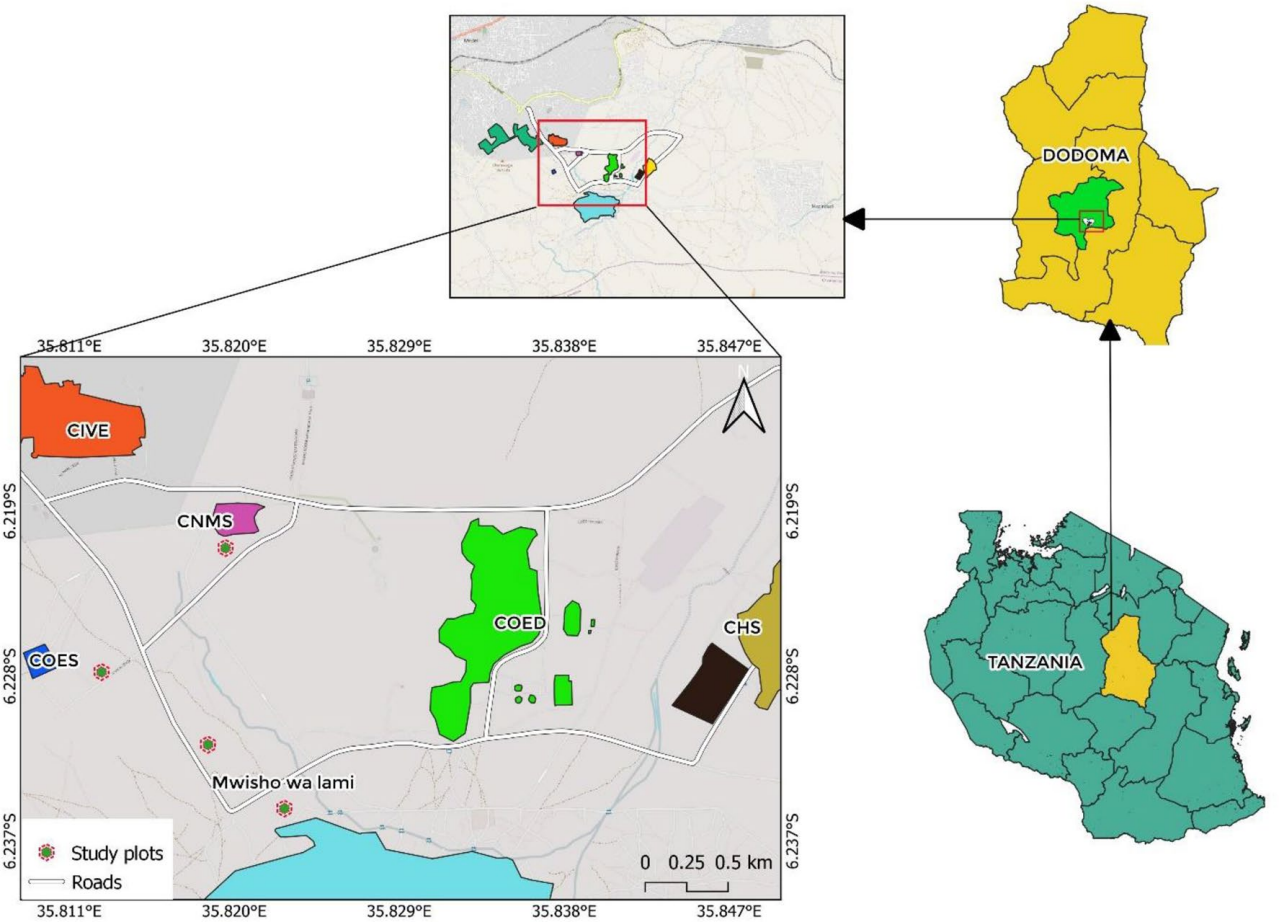
Map showing the study area within the University of Dodoma (Created using QGIS 3.28.0 Firenze version, 2022). Note: CNMS-College of natural and mathematical science, CHS-College of humanities and social science, CIVE- College of informatic and virtual education, COED-College of Education.

The study was carried out at the University of Dodoma (UDOM) and specifically at College of Natural and Mathematical Sciences (CNMS) and College of Education (COED) (Fig. 1). These two sites were considered because they have both afforested and non-afforested areas. Furthermore, unlike other places where afforestation is uncoordinated, the selected study area has proper management and records for the afforestation program that is taking place. The study area is located at latitude of 6° 57´ and 3° 82´ and longitudinal of 36° 26´ and 35° 26´. Its elevation is estimated to be 1120 m above the sea level. The site is semi-arid area dominated by sandy loam soil classified as Oxisol. The average annual rainfall of the areas is 447 mm. Temperatures vary depending on the season, with average minimum and maximum of $18^\circ{\rm C}$ and 32 $^\circ{\rm C}$ respectively.

The bush is leafless and dry during the dry season, but comes to life during the rainy season, when the entire countryside turns a vibrant green^19,20^ The remaining land is covered in woodlands, with the highest concentrations in hills (URT 2014). The vegetation consists of dry savanna shrub-thicket areas with scattered trees and grassland patches interrupted by trees and shrubs.

### Study design on abundance and diversity of lizards

Data on lizard abundance and diversity were collected at two sites, namely the CNMS and another site located at COED. These areas were purposely selected because the afforestation program is taking place. In the selected areas, trees have been planted for the past three years, which are 2019–2021. More effort is being made to plant more trees. Also, the areas have natural vegetation characterized by thickets, shrubs, and nature trees with species as described above in the study area. This makes the areas ideal for making comparisons between the afforested and un-afforested areas. In each site, two blocks were established, in which one block consisted of an afforested area while the other block was a non-afforested area.

### Data collection

#### Documentation of planted tree species

The plants observed in the study areas were recorded. In addition to that, we worked with the restoration team, which provided the list of tree species that are grown in those study areas. Secondary data was collected from the restoration team regarding the tree species and how much has been planted in the last 5 years in the study areas.

#### Sampling of lizard for abundance and diversity determination

##### Pitfall traps

Each block had a size of 60 m by 60 m (2600 m^2^). In each block, two transects were established, each with a length of 60 m and a spacing of 20 m. In each transect, 4 points were identified, whereby 10 pitfall traps of 5 L each were set at an interval of 12 m. This makes 40 pitfall traps and eight walking transects. Emptying was performed every morning for 10 consecutive days in each pitfall. Thus, a total of 800 samples were collected from pitfall traps, with 400 samples being collected at each site.

##### Direct searching

General direct searching involving time-constrained observation was also used to collect data on the lizards found in the study area. Time constrained searches were conducted as an opportunistic means of finding animals hiding under cover and flushing them as the observer approached. Searching was conducted in an area of 20 m × 20 m at each sampling point where pitfalls were set. Searching was performed by an individual who is an ecologist and is an expert in reptiles for 10 min, 3 times a day for 10 days (n = 240). To ensure consistency, the same individual was employed in searching for each sampling point.

At each site, the observed lizards were identified by their numbers and habitats. Photographs of captured or observed animals were taken to aid in identification. In addition, human activities such as cultivation, roads, tree cutting, building, and distance from roads and buildings, were recorded. Furthermore, more physical structures like rocks and distances from rocks were recorded. Identification of species of lizards was performed using a guide book for east African reptiles^21^.

#### Sampling and interview for the assessment of awareness of the importance of afforestation

A cross-sectional survey using a semi-structured questionnaire was used to collect data from undergraduate students in four colleges, which are CNMS, COED, CHSS, and CIVE. The respondents were selected randomly from each college. These students were selected based on their familiarity with the areas that are anticipated to see what is taking place within the University of Dodoma. It was anticipated that awareness would vary by college because the programs offered differed. For example, it was predicted that students from CNMS would be more aware than others because they have programs and courses that teach conservation, restoration, and afforestation knowledge. Both genders were included in the survey. A total of 394 interviewees were recruited; 100 participants were from CHSS, 103 from CIVE, 101 from CNMS, and 90 from COED. The questionnaire consisted of both closed and open-ended questions. The questions consisted of information on the demographic structure of students and their awareness of the afforestation program. Concerning awareness, the questions focused on their understanding of afforestation, their participation, and other stakeholders involved in the program.

Some questions had to be ranked from 1 to 5, with the answers classified as very high, high, moderate, low, and very low if they scored 5, 4, 3, 2, and 1, respectively. The questions were designed to elicit responses from respondents regarding their knowledge of the ongoing afforestation program. In addition, information on the program's participants and their level of involvement was requested.

#### Human ethical guideline statement

All methods were carried out in accordance with relevant guidelines and regulations.

### Ethical approval and consent to participate

The ethics committee of University of Dodoma granted ethical approval for this study, with reference number MA.84/261/02.

### Informed consent

Informed consent was obtained from all participants included in the study.

## Data analysis

The responses of interviews were summarized in tables. The variation/difference in frequencies of responses among colleges was determined using Chi square (χ^2^) because the data were not normally distributed (Shapiro–Wilk test = 0.75, *P* = 0.03). The Shapiro–Wilk test to test normality of data was performed using PAS software version 4.03 (Paleontological statistical software for education and data analysis)^22^. The association between the demographic structures (gender, age, college belonging, year of study) and their awareness (knowledge) and willingness to participate in afforestation was determined using a generalized linear mixed effect modal (GLMM) through R statistical software under "lme4" version 1.1-29 under the binomial family because of binomial responses (yes or no). In GLMM, demographic structures such as gender, age, education level, college, and marital status were the fixed effects, while awareness and willingness to be involved in afforestation programs were dependent variables, and the program of study was the random effect. The diversity of lizards in two vegetation types was determined using Shannon Wiener (H), Margalef, and Equitability (J).

## Results

### Afforested and non-afforested

The non-forested area comprised the native plants species which are found in Dodoma. The common native species includes the following: *Brachystegia spiciformis, Bussea massaiensis, Commiphora coerulea, C. ugogensis, Sclerocarrya birrea, C. africana, Acacia tortilis, A. senegal, Maerua decumbens, Grewia forbesii, Julbernardia globiflora, Delonix elata, Markhamia acuminate, Combretum apiculatum, Euphorbia candelabrum, and Terminalia sericea* as indigenous taxa, mixed with exotics such as *Peltophorum pterocarpum and Tamarindus indica*^19,20^*.* The afforested area comprised more than nine tree species. For three years, more than 25,000 trees have been planted. More species being planted in 2019 compared to other time (Table 1).

**Table 1:** Tree species planted in UDOM for afforestation program in three years (2019–2021).

Year	Tree planted for the past 3Years (2019–2021)	Species of tree planted	CHSS	CNMS	CIVE	COED
2019	12, 520	*Ashock*	X	Y	X	Y
		*Gliricidia sepium*	Y	X	X	X
		*Mangifera indica*	Y	X	X	X
		*Eucalyptus spp*	Y	X	X	X
		*Afzelia spp*	Y	X	X	X
		*Khaya anthoteca*	Y	X	X	X
		*Trichilia emetica*	Y	X	X	X
		*Cedrela audorata*	Y	X	X	X
		*Casuarina spp*	Y	X	X	X
		*Zyzigium cuminii*	Y	X	X	X
2020	1,500	*Mangifera indica anthoteca*	Y	X	X	X
		*Trichilia emetic*a	Y	X	X	X
		*Cedrella audorata*	Y	X	X	X
		*Casuarina spp*	Y	X	X	X
2021	11,400	*Mangifera indiaca*	Y	X	X	X
		*Citrus lemon*	Y	X	X	X
		*Anona muricata*	Y	X	X	X
		*Punica granatum*	Y	X	X	X
		*Persea Americana*	Y	X	X	X
		*Guava sativa*	Y	X	X	X

*CNMS*, college of natural and mathematical science; *CHSS*, college of humanities and social science; *CIVE*, college of informatic and virtual education; *COED*, college of education; *Y*, planted species; *X*, not planted.

### Awareness of respondents on afforestation program

A total of 394 people were interviewed, of whom 25.63% (n = 101) were from CNMS, 20.38% (n = 100) from CHSS, 26.14% (n = 103) from CIVE, and 22.84% (n = 90) from COED. The demographic structure of respondents varied insignificantly among the colleges. For instance, in CNMS, single males aged between 21 and 35 years old, mostly being first years, were the dominant group interviewed (Table 1). CIVE had similar gender, age group, and marital status trends as CHSS and COED, where the majority were female (Table 2).

**Table 2:** Demographic structure of respondents across the four colleges.

s/n	Variables	Classification	CNMS (n = 101)	CHSS (n = 100)	CIVE (n = 103)	COED (n = 90)	χ^2^	DF	*P*-value
1	Sex	Male	60	45	56	35	9.80	3	0.02
		Female	41	55	47	55			
2	Age group	18–20	27	11	20	11	14.19	6	0.03
		21–35	72	89	81	76			
		35–60	2	0	2	3			
3	Marital status	Married	18	21	29	42	21.08	3	< 0.001
		Single	83	89	55	58			
4	Year of Study	I	15	11	21	12	16.32	6	0.01
		II	30	46	49	38			
		III	56	37	31	40			
4	Program	Bsc. Bio	21	0	0	0	118.46		< 0.001
		Bsc AA	10	0	0	0			
		BSc BB	6	0	0	0			
		Non-biological program	64	100	103	90			

*P-value*, probability value; *CNMS*, college of natural and mathematical science; *CHSS*, college of humanities and social science; *CIVE*, college of informatics and virtual education; *COED*, college of education; *χ*^2^, Chi square; *D.F*., degree of freedom.

Across the four colleges, more than 95.94% (n = 378) of respondents acknowledged that they understood the afforestation program taking place within the university. However, 43.15% (n = 110) were not aware of who was responsible for the restoration program. The involvement of respondents in the afforestation program was observed to be 48.22% (n = 122). These responses varied insignificantly across the four study colleges (Table 2). Negative associations were observed between awareness and the age group of participants, while positive associations were observed for participants from the college of CNMS and third-year students (Table 3). There is a positive likelihood that students from CNMS are more aware of being involved in the afforestation program (Table 4).

**Table 3:** Awareness on the afforestation program across the study colleges.

s/n	Variables	Classification	CNMS (n = 101)	CHSS (n = 100)	CIVE (n = 103)	COED (n = 90)	χ^2^	D.F	*P*-value
1	Do you know about afforestation program?	Yes	93	99	97	89	9.10	3	0.03
		No	8	1	6	1			
2	Did you involve in afforestation program?	Yes	61	49	40	40	10.17	3	0.02
		No	40	51	63	50			
3	Do you know who is involved?	Yes	61	71	41	51	20.88	3	0.001
		No	40	29	62	39			
4	Name the one who is involved in afforestation program to UDOM community	TFS	5	0	4	21	109.43	9	< 0.001
		UDOM	40	4	13	5			
		UDOSO	7	25	14	11			
		Others	49	71	73	53			

*P-value*, probability value; *CNMS*, college of natural and mathematical science; *CHSS*, college of humanities and social science; *CIVE*, college of informatics and virtual education; *COED*, college of education; *χ*^2^, Chi square; *D.F*., degree of freedom.

**Table 4:** The association of awareness and the involvement of respondents with their gender, age, marital status, colleges their belong and years of study.

	Fixed effects						Random effects
	Variable	Classification	Coeff, Estimate	S. E	z value	*P*-value	
Awareness		Intercept	6.48	1.63	3.98	7.01e−05***	Random effect = Program, AIC = 137.0, BIC = 180.7 LogLik =− 57.5
	Gender	Male	− 0.54	0.58	− 0.93	0.36	
	Age (Years)	21–35	− 2.52	1.22	− 2.07	0.04*	
		35–60	− 3.94	1.80	− 2.19	0.03*	
	Marital status	Single	− 0.19	0.69	− 0.28	0.78	
	Colleges	CIVE	− 2.04	1.12	− 1.82	0.07	
		CNMS	− 2.66	1.13	− 2.36	0.02*	
		COED	− 0.29	1.44	− 0.20	0.84	
	Year of Study	II	1.05	0.83	1.27	0.21	
		III	1.79	0.90	1.997	0.04*	
Involvement		Intercept	− 2.30	0.83	− 2.78	0.01**	Random effect = Program, AIC = 543.8, BIC = 591.5, LogLik =− 259.9
	Gender	Male	− 0.19	0.22	− 0.90	0.37	
	Age (Years)	21–35	0.45	0.34	1.33	0.18	
		35–60	1.38	0.85	1.63	0.10	
	Awareness	Yes	1.75	0.68	2.59	0.01**	
	Marital status	Single	− 0.002	0.26	− 0.01	0.99	
	Colleges	CIVE	− 0.26	0.29	− 0.89	0.37	
		CNMS	0.70	0.31	2.26	0.02*	
		COED	− 0.18	0.30	− 0.60	0.55	
	Year of Study	II	0.10	0.34	0.29	0.77	
		III	0.29	0.36	0.82	0.41	

*P-value*, probability value; *S. E.*, standard error; *CNMS*, college of natural and mathematical science; *CHSS*, college of humanities and social science; *CIVE* college of informatics and virtual education; *COED*, college of education.

Significant effects were considered when *P* < 0.05, *-Significant effects seen.

### Diversity of lizards in the afforested areas and no-afforested

A total of 1040 samples (800 from pitfalls and 240 from direct search) were collected in the study area. Of which 50% (n = 520) were from forested areas, the other 50% (n = 520) were from non-forested areas. A total of lizards was recorded, namely eight species. The highest abundance of lizards was observed in *Agama lionotus,* followed by *Hemidactylus brooki*, then *Heliobolus spekiii* (5), *Agama armata*, *Hamidactylus mabouia*, *Chamaeleo dilepis*, *Trachylepis varia*, and *Trachylepis planifrons*. The forested area had a higher number of species (n = 7) compared to the unforested area (n = 2). Also, the abundance of lizards was observed to be higher in the forested area (n = 69) than in the non-forested area (n = 4) (Table 5). The Shannon, Wiener, and Margalef indices revealed that afforested areas had greater diversity than non-afforested areas (Table 5).

**Table 5:** Species of Lizard in afforested and non-afforested areas.

s/n	Common name	Scientific name	Afforested	Not-afforested
1	Kenyan rock agama	*Agama lionotus*	36	0
2	Brook’s gecko	*Hemidactylus brooki*	19	5
3	Speke's sand lizard	*Heliobolus spekiii*	5	0
4	Peter's ground agama	*Agama armata*	4	2
5	Tropical house gecko	*Hamidactylus mabouia*	4	0
6	Flat necked chameleon	*Chamaeleo dilepis*	0	2
7	Variable Skink	*Trachylepis varia*	2	0
8	The tree skink	*Trachylepis planifrons*	1	0
9		Total	71	9
		P-value	0.001	
10	Diversity indices	Shannon_H	1.37	0.99
		Evenness_e^H/S	0.56	0.90
		Margalef	1.41	0.91
		Equitability_J	0.70	0.91

## Discussion

### The afforestation and ecosystem services

Tree planting is critical for the health of Dodoma's ecosystem. The trees planted in the study area are well adapted to semi-arid environments such as Dodoma. Afforestation has the potential to improve ecosystem services in a target area, as was observed for lizards in this study and insects such as beetles^16^. Ecological and social benefits are included in the enhancement of ecosystem services. The social benefits include the modification of the microclimate, the provision of shade, firewood, and poles made from dead wood. Other wildlife species, such as lizards, insects, birds, and other mammals, benefit from ecosystem services. Other reports have highlighted additional benefits of afforestation, such as overcoming climate change challenges, controlling soil erosion and flooding, and producing oxygen through the photosynthesis process^23^. Other findings have shown that afforestation has the potential to advance the ecosystem into a more complex state, which will have a positive impact on the ecosystem^24^.

### Awareness of respondents on afforestation program

Across the colleges, the dominant age group was young (18–20 years old). Other demographic structures differed between the study colleges. For example, in CNMS, the majority of respondents were male, whereas in other colleges, females predominated. Gender, age, conservation, and afforestation strategies must all be considered. In Tanzania, most men work in the charcoal and lumber industries, while women are known to harvest trees for firewood and other domestic purposes. All of these activities are harmful to the long-term viability of forested areas. For example, a study in Dar-es-Salaam discovered that charcoal production, which is frequently dominated by men^25^.

Respondents studying natural sciences demonstrated greater awareness than other participants. Conservation and ecological skills are among the training they receive in their program, which includes biology, forest, and aquaculture courses. This study found that awareness was positively related to the likelihood of an individual participating in an afforestation program. This implies that if the communities are most aware of the afforestation program, they will be willing to be involved in the afforestation and conservation efforts of the afforested. One of the afforestation components is the best conservation planning for the afforested area. Afforestation and conservation go hand in hand. This means that forested areas require proper conservation efforts that include the surrounding communities. Training has been reported to increase local community awareness and participation in afforestation and conservation efforts^26^. Furthermore, a study from Tanzania's Magombera forest reserve found that alternative livelihood and conservation education motivated local communities to participate in forest restoration and conservation^10^. Other Tanzanian studies have found similar effects of conservation education and awareness on biodiversity conservation^9,27^.

### Diversity of lizards in the afforested areas

Higher diversity and abundance in forested areas compared to non-forested areas can be attributed to changes in habitats that support lizard species in terms of shade, food, and shelter. The higher abundance and diversity of lizards in the afforested area can be associated with the age of the forest and species of trees involved in afforestation. The study area had grown by 5 years since restoration began, and some species were new to the area, such as *Mangifera indica* and *Citrus lemon.* Relating findings were observed in the study area, on beetles where age of afforestation showed impacts on abundance and species diversity^16^. The difference in abundance and diversity of lizard species between the two vegetation structures indicates that lizards are sensitive to changes in their habitat or ecosystem. In this case, they can serve as good indicators for the ecosystem as well as other afforestation and restoration efforts being undertaken by various stakeholders. An indicator species, such as lizards, is one whose presence, absence, or abundance reflects a specific environmental condition. Indicator species can signal a change in the biological condition of a specific ecosystem and thus serve as a proxy for detecting the health of that ecosystem. ^17^ discovered that lizards are good indicator species in arid areas. This lends support to the use of lizards in semi-arid regions of Tanzania such as Dodoma, where other species such as amphibians are scarce during the dry season.

## Conclusion and recommendation

Afforestation in Dodoma is a good idea for both environmental and social reasons. It is clear from this study that afforestation can be improved and potential ecological and social benefits realized if local communities' awareness is raised. This implies that more conservation education for local communities surrounding afforestation areas is required. Furthermore, monitoring the succession of the restored areas is critical for successful afforestation. This study demonstrated that using sensitive species such as lizards can be very useful in understanding the progress of restored areas. Lizard abundance and diversity differed between the two categories of sites in this case, which are afforested areas and non-afforested areas.

## Data Availability

The datasets used and/or analyzed during the current study available from the corresponding author on reasonable request.
